# Supplementary material for: Relationship of insect biomass and richness with land use along a climate gradient
Source: Nat Commun. 2021 Oct 12;12:5946. doi: 10.1038/s41467-021-26181-3 (PMC8511018; doi:10.1038/s41467-021-26181-3)
Supplement: Supplementary file 1 — Supplementary Information [file 41467_2021_26181_MOESM1_ESM.pdf]

Supplementary Information for

Relationship of insect biomass and richness with land use along a climate gradient

Johannes Uhler[1], Sarah Redlich[2], Jie Zhang[2], Torsten Hothorn[3], Cynthia Tobisch[4], Jörg Ewald[5], Simon Thorn[1], Sebastian Seibold[6,7], Oliver Mitesser[1], Jérôme Morinière[8], Vedran Bozicevic[8], Caryl S. Benjamin[9], Jana Englmeier[1], Ute Fricke[2], Cristina Ganuza[2], Maria Haensel[10], Rebekka Riebl[10], Sandra Rojas Botero[11], Thomas Rummler[12], Lars Uphus[9], Stefan Schmidt[13], Ingolf Steffan-Dewenter[2], Jörg Müller[1,14]*

*corresponding author: joerg.mueller@npv-bw.bayern.de

Supplementary Information:

Supplementary Figures 1-4

Supplementary Tables 1-3

Supplementary Methods

Supplementary References (1-12)

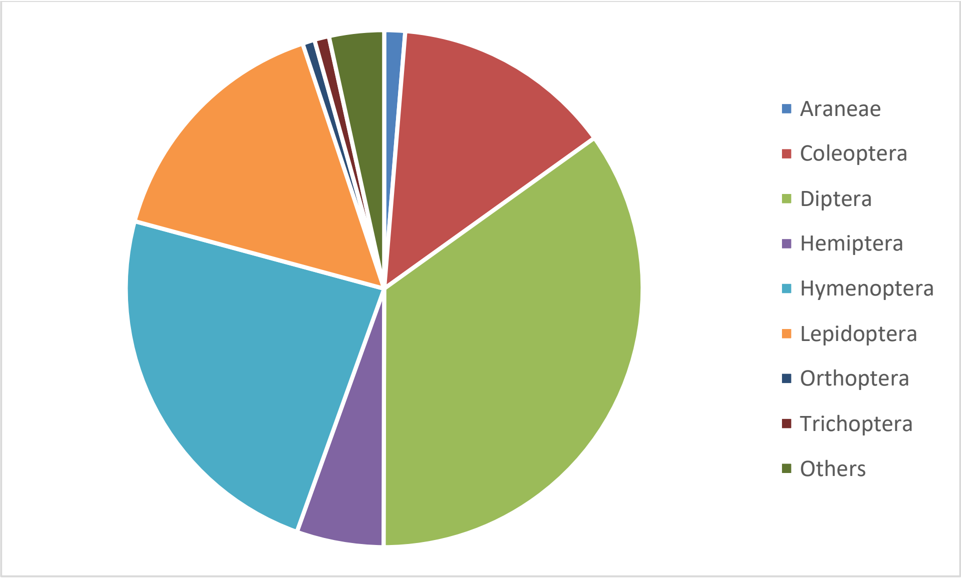

Supplementary Figure 1. Distribution of detected BINs across taxonomic orders. Only the eight most common orders are displayed, the remainder was combined in the category "Others".

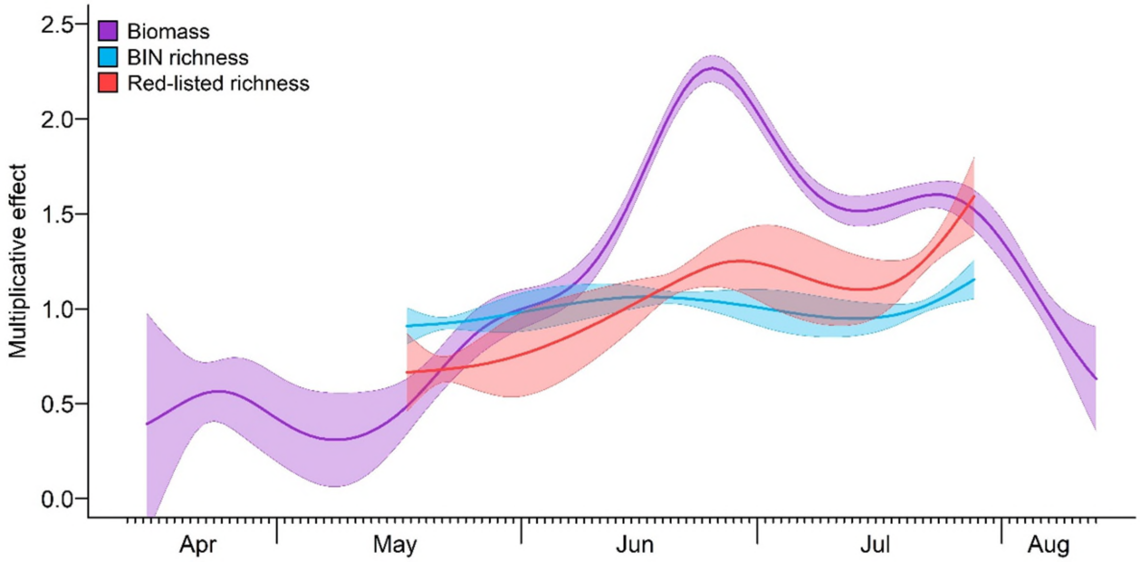

Supplementary Figure 2. Partial effects of season on biomass, total richness of barcode index numbers (BINs), and the richness of red-listed species without correcting for local temperature and humidity. Partial effects from generalized additive mixed models were controlled for elevation, the geographic location of the traps, and land use. Note that richness was determined for only three of the eight

sampling campaigns. Displayed are the partial effect of season, as a smooth term acting multiplicatively on the expected outcome per time unit**.** Error envelopes depict standard errors below and above the estimated mean responses.

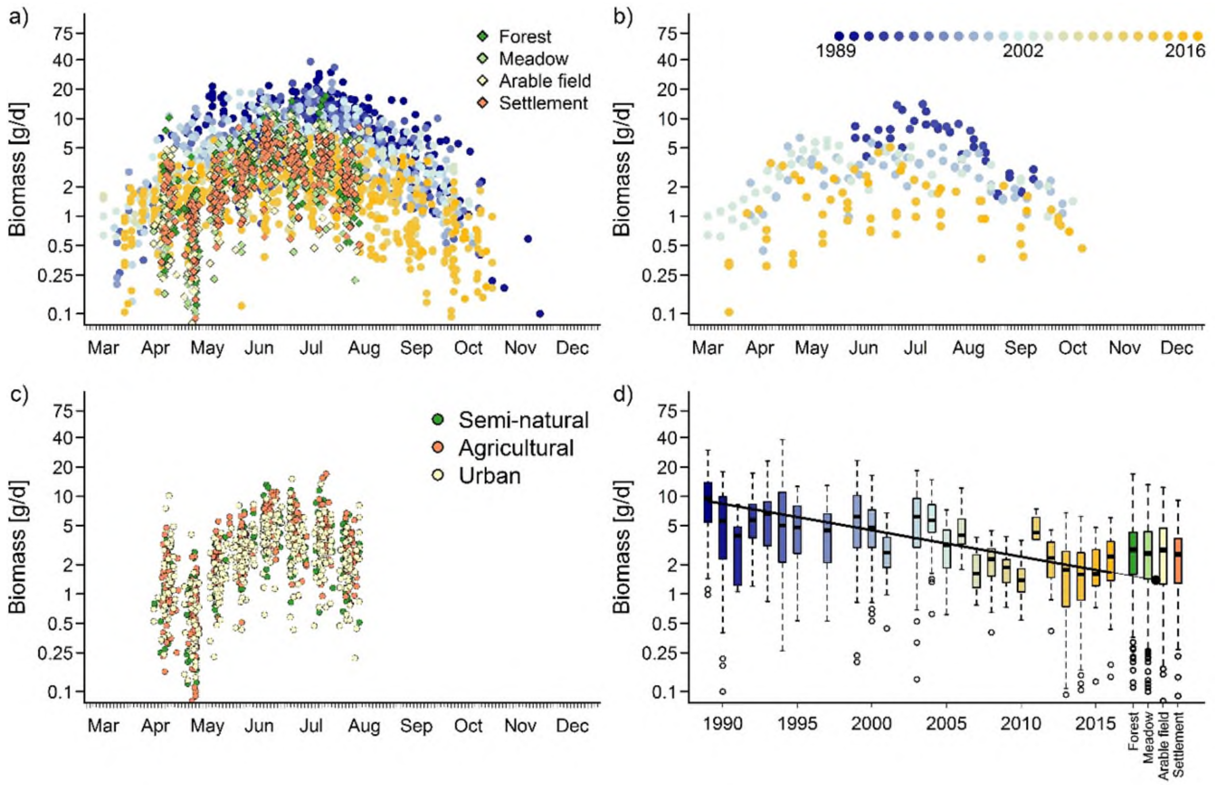

Supplementary Figure 3. Comparison with biomass data reported in Hallmann et al.[12]. Hallmann et al.[12] data were collected with a similar malaise trap type in protected areas of Northern Germany. a) all data points from Hallmann et al.[12] in yellow, pale blue and dark blue throughout the season and LandKlif data colored according to local landuse type. b) semi-natural plots in Hallmann et al.[12] (here plotted separately) showed similar patterns in time as the other habitats. c) all LandKlif data points throughout the season by landscape type. d) long-term trends over 27 years (Hallmann et al.[12] , n=1503; linear slope extrapolated to continue line to 2019 for visual reference) and LandKlif data points by local habitat (2019, n=1293). The black lines of the box plots show the medians, boxes represent data within the 25th and 75th percentile and whiskers display 1.5x the interquartile range.

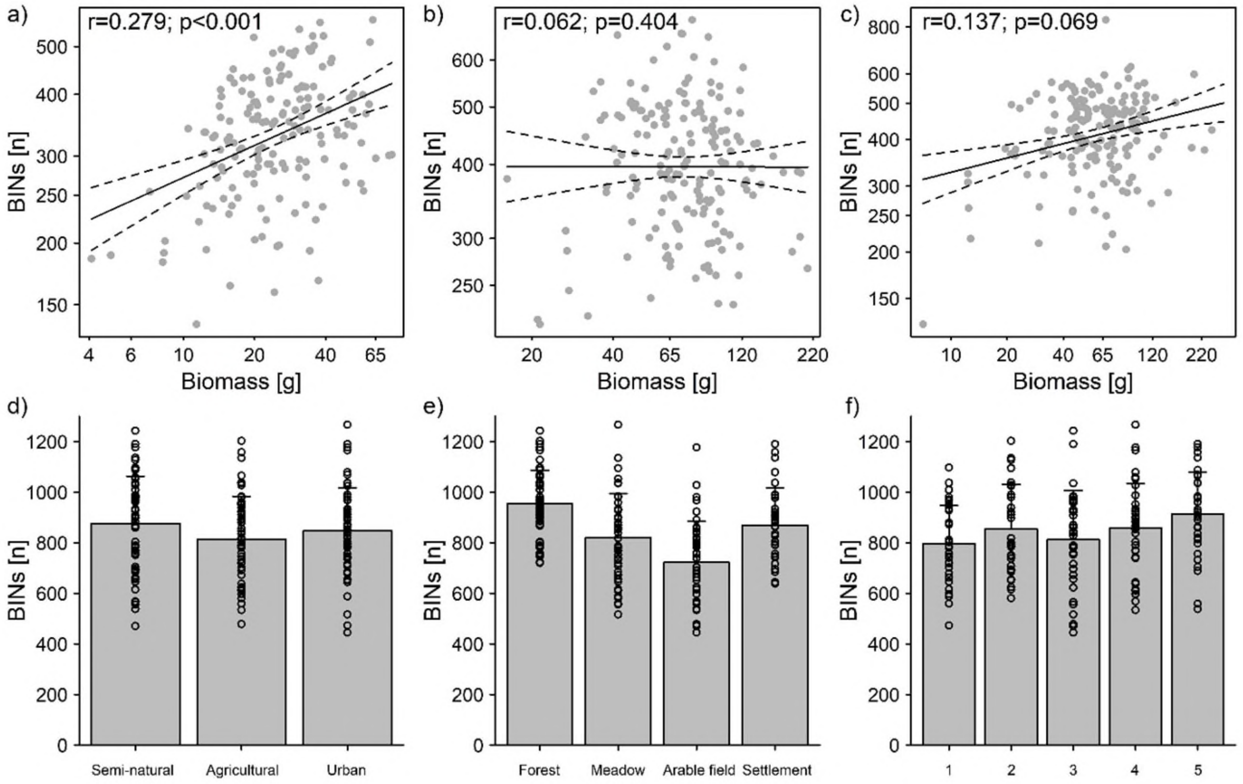

Supplementary Figure 4. Correlation plots for Biomass and BINs for the three sampling campaigns in May (a), June (b), July (c) and accumulative number of BINs across habitat (d), landscape (e) and climate categories (f) for all 179 study sites. The continues black line in a)-c) represents the linear regression line with confident intervals displayed as dashed lines. Climate zones were defined based on the mean annual temperature over 30 years (1981–2010): <7.5, 7.5–8, 8–8.5, 8.5–9, >9°C, see also method section. Data in d)-f) is shown as mean values (n=179), error bars display standard deviation.

Supplementary Table 1. Name and Sequence of Primers used for multiplex PCR. HTS-adapted mini-barcode primers targeting mitochondrial CO1-5P region

Primers	Sequence
mlCOIintF	GWACWGGWTGAACWGTWTAYCCYCC
dgHCO2198	TAAACTTCAGGGTGACCAAARAAYCA

Supplementary Table 2. Results of a generalized additive mixed model for average local temperature. Values are displayed in comparison to local forests for local habitat scale and to semi-natural landscapes for landscape scale categories. For additional model parameters see Table 1. For additional information see annotated code.

	Local Temperature			
Predictors	Estimates * 10^3	std. Error * 10^3	t-value	p
(Intercept)	17194.9	1739.5	9.885	**<0.001**
Local land use: Forest				
Meadow	316.2	72.22	4.379	**<0.001**
Arable field	494.0	74.05	6.671	**<0.001**
Settlement	738.9	81.35	9.084	**<0.001**
Landscape land use: Semi-natural				
Agricultural	-51.59	91.73	-0.562	0.573
Urban	198.77	94.30	2.108	**0.035**
Long-term mean annual precipitation	-2.396	0.700	-3.423	**<0.001**
Long-term mean annual near surface temperature	164.5	138.0	1.192	0.233
Observations	1301			
R^2	0.957			

Supplementary Table 3. Number of excluded samples per landscape and habitat type: Overall, 142 samples were excluded from the statistical analyses, 46 due to missing climate data and 96 due to other complications. This resulted in the exclusion of 139 samples from the analysis of the biomass and 27 for the analysis of BINs.

	**Landscape type:** Semi-natural	Agricultural	Urban
**Habitat type:**			
Forest	17	17	13
Meadow	13	4	10
Arable field	4	26	9
Settlement	4	1	24
**Total:**			**142**

Supplementary Methods

Next generation sequencing

Preservative ethanol was removed and the mixed arthropod samples were dried overnight in a 60–70°C oven to evaporate off the residual ethanol. The dried arthropods were then homogenised with stainless steel beads within a FastPrep 96 system (MP Biomedicals). DNA was extracted from all samples by incubating them in a 90:10 solution of animal lysis buffer (buffer ATL, Qiagen DNEasy tissue kit, Qiagen, Hilden, Germany) and proteinase K. After an overnight incubation in a 56°C oven, the samples were left to cool to room temperature. DNA was extracted from 200-µL aliquots using the DNEasy blood & tissue kit (Qiagen) following the manufacturer's instructions. Multiplex PCR was performed using 5 µL of extracted genomic DNA, Plant MyTAQ (Bioline, Luckenwalde, Germany) and high-throughput sequencing (HTS)-adapted mini-barcode primers (Supplement Table1) targeting the mitochondrial CO1-5P region, following Leray et al., 2013[1] – also see Morinière *et al.*, 2016[2]; Morinière *et al.*, 2019[3].

Amplification success and fragment length were determined using gel electrophoresis. The amplified DNA was cleaned and each sample was resuspended in 50 µL of molecular water.

Illumina Nextera XT (Illumina Inc., San Diego, USA) indices were ligated to the samples in a second PCR, conducted at the same annealing temperature as in the first but with only seven cycles. Ligation success was confirmed by gel electrophoresis. DNA concentrations were measured using a Qubit fluorometer (Life Technologies, Carlsbad, USA), and the samples then combined into 40-µL pools containing equimolar concentrations of 100 ng each. The pooled DNA was purified using MagSi-NGSprep Plus beads (Steinbrenner Laborsysteme GmbH, Wiesenbach, Germany). The final elution volume was 20 µL. HTS was performed on an Illumina MiSeq using v3 chemistry (2*300bp, 600 cycles, maximum of 25mio paired-end reads).

Bioinformatics

Paired-ends were merged using the *-fastq_mergepairs* utility of the USEARCH suite v11.0.667_i86linux32[4] with the following parameters: *-fastq_maxdiffs* 99, *-fastq_pctid* 75, *-fastq_trunctail* 0. Adapter sequences were removed using CUTADAPT K5 (default parameters). All sequences that did not contain the appropriate adapter sequences were filtered out in this step using the *--discard-untrimmed* parameter. The remaining pre-processing steps (quality filtering, dereplication, chimera filtering, and clustering) were carried out using the VSEARCH suite v2.9.1[6]. Quality filtering was performed using the *--fastq_filter* VSEARCH utility (parameters: *--fastq_maxee* 1, *--minlen* 300). Sequences were dereplicated with *--derep_fulllength* (parameters: *--sizeout*, *--relabel Uniq*), first at the sample level (output: ***all.derep.uc***), and then at the combined dataset level after concatenating all sample files into one large FASTA file (***all.fasta***), which was also filtered for singletons (sequences occurring only once in the entire dataset and *a priori* considered as noise; parameters: *--minuniquesize* 2, *--sizein*, *--sizeout*, *--fasta_width* 0; resulting file: ***all.derep.fasta***). To save processing power, a pre-clustering step (at 98% identity) was employed before chimera filtering using the *--cluster_size* VSEARCH utility with the centroids algorithm (parameters: *--id* 0.98, *--strand plus*, *--sizein*, *--sizeout*, *--fasta_width* 0, *--centroids*; input: *all.derep.fasta*; outputs: ***all.preclustered.uc***, ***all.preclustered.fasta***). Chimeric sequences were then detected and filtered out from the resulting file using the VSEARCH *--uchime_denovo* utility (parameters: *--sizein*, *--sizeout*, *--fasta_width* 0, *--nonchimeras*; input: *all.preclustered.fasta*; output: ***all.denovo.nonchimeras.fasta***). The remaining sequences were then clustered into OTUs at 97% identity using *--cluster_size* (parameters: see below).

To create the OTU table, a custom perl script was used to extract all non-chimeric non-singleton sequences from the dereplicated dataset (inputs: *all.derep.fasta*, *all.preclustered.uc*, *all.denovo.nonchimeras.fasta*; output: ***all.nonchimeras.derep.fasta***), and then all non-chimeric non-singletons from each sample (inputs: *all.fasta*, *all.derep.uc*, *all.nonchimeras.derep.fasta*; output: ***all.nonchimeras.fasta***). The task of perl script was to recover all of the quality- and chimera-filtered sequences from the individual samples, including singletons, as well as sequences that had been removed during the two rounds of dereplication. The resulting file (*all.nonchimeras.fasta*) was then used to map the reads to the OTUs and thus create the OTU table (parameters: --*cluster size* all.nonchimeras.fasta, --*id* 0.97, --*strand* plus, --*sizein*, --*sizeout*, --*fasta_width* 0, --*uc*, --*relabel* OTU, --*centroids* ***otus.fasta***, --*otutabout* ***otu_table.txt***). To reduce the risk of false-positives, a cleaning step was employed that excluded read counts in the OTU table constituting < 0.01% of the total number of reads in the sample. OTUs were blasted (parameters: program: Megablast; maximum hits: 1; scoring (match mismatch): 1-2; gap cost (open extend): linear; max E-value: 10; word size: 28; max target seqs 100) against (1) a custom database downloaded from GenBank (a local copy of the NCBI nucleotide database downloaded from ftp://ftp.ncbi.nlm.nih.gov/blast/db/), and (2) a custom database built from data downloaded from BOLD (www.boldsystems.org)[7,8] including taxonomy and BIN information, by means of Geneious (v.10.2.5 – Biomatters, Auckland, New Zealand), and following the methods described in Morinière *et al.* (2016)[2]. The resulting csv files, which included the OTU ID, BOLD Process ID, BIN, Hit-%-ID value (percentage of overlap similarity (identical base pairs) of an OTU query sequence with its closest counterpart in the database), Grade-%-ID value (combining query coverage, E-value and identity values for each hit with weights of 0.5, 0.25 and 0.25 respectively, allowing determination of the longest, highest-identity hits), the length of the top BLAST hit sequence, as well as the phylum, class, order, family, genus and species information for each detected OTU were exported from Geneious and combined with the OTU table generated by the bioinformatic pre-processing pipeline. As an additional measure of control other than BLAST, the OTUs were classified into taxa using the Ribosomal Database Project (RDP) naïve Bayesian classifier[9] trained on a cleaned COI dataset of Arthropods and Chordates (plus outgroups; see Porter & Hajibabei, 2018)10. To reduce the risk of false-positives, the combined results table was then filtered, excluding those read counts in the OTU table accounting for < 0.01% of the total number of reads in the sample. OTUs were additionally removed from the results based on negative

control samples, i.e. if the combined number of reads in the negative controls constituted > 20% of the total number of reads in the OTU. OTUs were also annotated with the taxonomic information from the NCBI (downloaded from https://ftp.ncbi.nlm.nih.gov/pub/taxonomy/), followed by the creation of a taxonomic consensus between BOLD, NCBI and RDP. Interactive Krona charts were produced from the taxonomic information using KronaTools v1.311.

Supplementary References

1. Matthieu, L. *et al.* A new versatile primer set targeting a short fragment of the mitochondrial COI region for metabarcoding metazoan diversity: application for characterizing coral reef fish gut contents. *Frontiers in Zoology* **10**, **2013**
2. Morinière, J. *et al.* Species Identification in Malaise Trap Samples by DNA Barcoding Based on NGS Technologies and a Scoring Matrix. *PloS one* **11**, e0155497 (2016).
3. Morinière, J. *et al.* A DNA barcode library for 5,200 German flies and midges (Insecta: Diptera) and its implications for metabarcoding-based biomonitoring. *Molec. Ecol. Resources* **19**, 900–928 (2019).
4. Edgar, R., C., Search and clustering orders of magnitude faster than BLAST. *Bioinformatics (Oxford, England)* **26**, 2460–2461 (2010).
5. Martin, M. Cutadapt removes adapter sequences from high-throughput sequencing reads. *EMBnet* (2011).
6. Rognes, T., Flouri, T., Nichols, B., Quince, C., Mahé, F., VSEARCH: a versatile open source tool for metagenomics. *PeerJ* **4**, e2584 (2016).
7. Ratnasingham, S., Hebert, P., D., N., bold: The Barcode of Life Data System (http://www.barcodinglife.org). *Molec. Ecol. Resources* **7**, 355–364 (2007).
8. Ratnasingham, S., Hebert, P., D., N., A DNA-based registry for all animal species: the barcode index number (BIN) system. *PloS one* **8**, e66213 (2013).
9. Wang, Q., Garrity, G., M., Tiedje, J., M., Cole, J., R., Naive Bayesian classifier for rapid assignment of rRNA sequences into the new bacterial taxonomy. *Appl. Environ. Microb.* **73**, 5261–5267 (2007).
10. Porter, T., M., Hajibabaei, M., Automated high throughput animal CO1 metabarcode classification. *Sci. Rep.* **8**, 4226 (2018).
11. Ondov, B., D., Bergmann, N., H., Phillippy, A., M., Interactive metagenomic visualization in a Webbrowser. *Bioinformatics* **2011.**
12. Hallmann, C. A. *et al.* More than 75 percent decline over 27 years in total flying insect biomass in protected areas. *PloS one* **12,** e0185809; 10.1371/journal.pone.0185809 (2017).
