# Supplementary material for: Relationship of insect biomass and richness with land use along a climate gradient
Source: Nat Commun. 2021 Oct 12;12:5946. doi: 10.1038/s41467-021-26181-3 (PMC8511018; doi:10.1038/s41467-021-26181-3)
Supplement: Supplementary file 3 — Reporting Summary [file 41467_2021_26181_MOESM3_ESM.pdf]

nature portfolio

Corresponding author(s): Jörg Müller

Last updated by author(s): Aug 26, 2021

Reporting Summary

Nature Portfolio wishes to improve the reproducibility of the work that we publish. This form provides structure for consistency and transparency in reporting. For further information on Nature Portfolio policies, see our Editorial Policies and the Editorial Policy Checklist.

Statistics

For all statistical analyses, confirm that the following items are present in the figure legend, table legend, main text, or Methods section.

n/a	Confirmed	
☐	☒	The exact sample size (*n*) for each experimental group/condition, given as a discrete number and unit of measurement
☐	☒	A statement on whether measurements were taken from distinct samples or whether the same sample was measured repeatedly
☐	☒	The statistical test(s) used AND whether they are one- or two-sided<br>*Only common tests should be described solely by name; describe more complex techniques in the Methods section.*
☐	☒	A description of all covariates tested
☐	☒	A description of any assumptions or corrections, such as tests of normality and adjustment for multiple comparisons
☐	☒	A full description of the statistical parameters including central tendency (e.g. means) or other basic estimates (e.g. regression coefficient) AND variation (e.g. standard deviation) or associated estimates of uncertainty (e.g. confidence intervals)
☐	☒	For null hypothesis testing, the test statistic (e.g. *F*, *t*, *r*) with confidence intervals, effect sizes, degrees of freedom and *P* value noted<br>*Give P values as exact values whenever suitable.*
☒	☐	For Bayesian analysis, information on the choice of priors and Markov chain Monte Carlo settings
☐	☒	For hierarchical and complex designs, identification of the appropriate level for tests and full reporting of outcomes
☐	☒	Estimates of effect sizes (e.g. Cohen's *d*, Pearson's *r*), indicating how they were calculated

Our web collection on statistics for biologists contains articles on many of the points above.

Software and code

Policy information about availability of computer code

Data collection	No software was used to collect the data
Data analysis	Data analyses were performed using R v.3.6.2. Generalized additive models were fitted using the package mgcv. Multiple post-hoc comparisons were done using the package multcomp. Linear mixed models were performed using the package lme4. Other packages used i.e. for drawing the graphs: sjPlot, scales, plotrix, jpeg, imager, rvest, knitr, corrplot.

For manuscripts utilizing custom algorithms or software that are central to the research but not yet described in published literature, software must be made available to editors and reviewers. We strongly encourage code deposition in a community repository (e.g. GitHub). See the Nature Portfolio guidelines for submitting code & software for further information.

Data

Policy information about availability of data

All manuscripts must include a data availability statement. This statement should provide the following information, where applicable:

- Accession codes, unique identifiers, or web links for publicly available datasets
- A description of any restrictions on data availability
- For clinical datasets or third party data, please ensure that the statement adheres to our policy

Raw data from the malaise trap samples including environmental data are publicly available from Dryad (https://doi.org/10.5061/dryad.zkh1893bb). An annotated R code including the data needed to reproduce the statistical analyses is also publicly available from Dryad (https://doi.org/10.5061/dryad.zkh1893bb).

Field-specific reporting

Please select the one below that is the best fit for your research. If you are not sure, read the appropriate sections before making your selection.

- [ ] Life sciences
- [ ] Behavioural & social sciences
- [x] Ecological, evolutionary & environmental sciences

For a reference copy of the document with all sections, see nature.com/documents/nr-reporting-summary-flat.pdf

Ecological, evolutionary & environmental sciences study design

All studies must disclose on these points even when the disclosure is negative.

Study description	We studied arthropod communities collected by Malaisetraps and identified by Metabarcoding along a land use and climate gradient. Overall, 179 Malaise traps were set up in 2019 along a gradient of increasing intensity, ranging from forests, to meadows, to arable fields, and settlements. Malaise traps are versatile passive net-traps, capable of catching a large spectrum of insect taxa and is very effective when the aim is to get a representative snapshot of the local insect community. The analyzed sites were spatially distributed over 400km and covered an elevational gradient of 1000m. Generalized additive models were fitted to test for the effects of local and landscape-level land-use categories on insect biomass and species richness.
Research sample	An insect sample in this study is the total amount of insects caught by a Malaisetrap at a given site over the course of two weeks and is considered representative for the insect community at the respective study site. Overall, we collected 1,293 insect samples. From the meta-barcoding results of 510 samples, 7589 BINs were identified. A Dataset from another study using Malaise traps, Hallmann et al. (2017), was used as a comparison.
Sampling strategy	We collected data based on a stratified selected set of different habitats and climate conditions based on GIS analyses reported in Redlich et al (doi.org/10.1101/2021.03.05.434036). For meta-barcoding, samples of three time periods of increased insect acttivity (May, June and July) were selected.
Data collection	Insect samples were obtained using Malaise traps in the field weighed in the lab and subsequently meta-barcoded. All people involved are coauthors, or listed in the acknowledgment section.
Timing and spatial scale	Sampling took place from May to August 2019 on 179 plots all across Bavaria, Germany.
Data exclusions	No data was exluded from the analysis
Reproducibility	This study was conducted on 179 study sites in 2019. Repeating this experiment under similar (climatic) conditions will not be possible. All data used for analysis is publicly available and allows full reproducibility of all results and graphs.
Randomization	The sampling followed a strict a prior selection of sites only based on GIS information and with sufficient replicates Redlich et al (doi.org/10.1101/2021.03.05.434036)
Blinding	Blinding is not possible in setting up insect traps (doi.org/10.1101/2021.03.05.434036)

Did the study involve field work? [x] Yes [ ] No

Field work, collection and transport

Field conditions	Malaise traps are passive traps and operated in all weather conditions from April to August. We measured local temperature and moisture with data loggers. These data are fully presented in our data and included in analyses.
Location	The sampling plots are spread over the federal state Bavaria in South Eastern Germany. For further information regarding the study sites (e.g. coordinates) see the available data file.
Access & import/export	The material was collected with the permission of the nature conservation authorities of the governments Upper Franconia (17.04.2019), Lower Franconia (09.05.2019), Middle Franconia (19.03.2019), Lower Bavaria (10.05.2019), Upper Bavaria (15.05.2019), Swabia (12.03.2019), Upper Palatinate (02.05.2019).
Disturbance	Several traps collapsed, or were destroyed. See Supplementary table 3 for further information.

Reporting for specific materials, systems and methods

We require information from authors about some types of materials, experimental systems and methods used in many studies. Here, indicate whether each material, system or method listed is relevant to your study. If you are not sure if a list item applies to your research, read the appropriate section before selecting a response.

Materials & experimental systems

n/a	Involved in the study
☒	☐ Antibodies
☒	☐ Eukaryotic cell lines
☒	☐ Palaeontology and archaeology
☐	☒ Animals and other organisms
☒	☐ Human research participants
☒	☐ Clinical data
☒	☐ Dual use research of concern

Methods

n/a	Involved in the study
☒	☐ ChIP-seq
☒	☐ Flow cytometry
☒	☐ MRI-based neuroimaging

Animals and other organisms

Policy information about studies involving animals; ARRIVE guidelines recommended for reporting animal research

Laboratory animals	This study did not involve laboratory animals
Wild animals	Arthropods were captured with Malaise traps in the field using ethanol as trapping and killing medium. The identification and further analysis requires the killing of the arthropods.
Field-collected samples	The sampled Arthropods were preserved in 80% ethanol.
Ethics oversight	The material was collected with the permission and ethical approval of the nature conservation authorities of the governments Upper Franconia, Lower Franconia, Middle Franconia, Lower Bavaria, Upper Bavaria, Swabia, Upper Palatinate.

Note that full information on the approval of the study protocol must also be provided in the manuscript.
